# And say the AI responded? Dancing around ‘autonomy’ in AI/human encounters

**DOI:** 10.1177/03063127231193947

**Published:** 2023-08-31

**Authors:** Emma Dahlin

**Affiliations:** Stockholm University, Stockholm, SE, Sweden

**Keywords:** artificial intelligence, autonomy, nonhumans, agency, AI/human relations

## Abstract

The article explores technology-human relations in a time of artificial intelligence (AI) and in the context of long-standing problems in social theory about agency, nonhumans, and autonomy. Most theorizations of AI are grounded in dualistic thinking and traditional views of technology, oversimplifying real-world settings. This article works to unfold modes of existence at play in AI/human relations. Materials from ethnographic fieldwork are used to highlight the significance of autonomy in AI/human relations. The analysis suggests that the idea of autonomy is a double-edged sword, showing that humans not only coordinate their perception of autonomy but also switch between registers by sometimes ascribing certain autonomous features to the AI system and in other situations denying the system such features. As a result, AI/human relations prove to be not so much determined by any ostensive delegation of tasks as by the way in which AI and humans engage with each other in practice. The article suggests a theory of relationality that redirects focus away from questions of agency towards questions of what it means to be in relations.

It’s supposed to be automatic, but actually you have to push this button.

(Brunner, 1969)

Artificial intelligence (AI) and other advanced robotic technological systems are gradually becoming central to the coordination of areas such as governance (Dafoe, 2018), financial markets (Muniesa, 2014), and health care (Topol, 2019). Technological development is happening rapidly, and people are struggling to learn how to work with such technological innovations (Carboni et al., 2023; Chevallier, 2022; Dahlin, 2021; Henriksen & Blond, 2023). Haraway (1991) observes that, in the history of Western scientific culture, machines have not been autonomous or self-moving. In practice, as more technologies are being ascribed decision-making and control features, boundaries are being renegotiated around the notions of ‘machine’, ‘self’, and what it means to be ‘autonomous’. This, in turn, further elevates questions of technology/human relations in ways for which we do not yet have a theoretical or empirical understanding.

There are longstanding questions in social theory concerning nonhumans and agency. Grounded in human exceptionalism, dominant views have excluded nonhumans from social theory and have resisted nonhuman agency. This applies to prevailing theorizations of technology as much as for other nonhumans. Literature examining narratives of AI show that traditional views on technology affect how research is being done on AI, and how AI is being developed (Cave et al., 2020; Suchman & Weber, 2016). A majority of AI narratives are built around starkly divided hopes and fears about AI, pessimistic vs. optimistic views on it, or seeing AI as either controlling humans or being controlled by humans. This is problematic since such thinking oversimplifies real-world encounters between technology and humans. Such problems point to the need to rethink technology/human relations. Against such a background, this article poses the following question: What is the significance of autonomy in AI/human relations?

To answer this question, we turn to a real-world environment to explore what Haraway (2008) would refer to as ‘contact zones’ between AI and humans—zones in which AI and human relate and make their ‘partial connections’ (Strathern, 1991). In an effort to account for some of the ways in which AI and humans make their relations, the article empirically explores AI/human relations in medical practice. AI and robotic systems are developing rapidly in medicine, requiring medical staff to learn how to work and cooperate with such technology (Grace et al., 2018; Johnston, 2018; Norgeot et al., 2020). Specifically, the article draws on ethnographic materials from radiosurgery practice with the AI and robotic system CyberKnife (Accuray Incorporated, Sunnyvale, CA, USA).

This article cross-pollinates science and technology studies (STS) and multispecies studies for the purpose of rethinking ideas of autonomy, agency, and nonhumans (Haraway, 2018; Latour, 1996). Scholars in these fields have continuously worked to connect worlds that are commonly kept apart in science—a natural world (without humans) and a social world (without objects) (Latour, 2017)—attending to the entanglements of life and things while acknowledging their interdependence (Latour, 2000). I offer a contrast to multispecies works and their mainly biotic focus by highlighting how abiotic beings affect and shape what Jasanoff (2021) calls ‘world-making practices’. I also add to work done in STS on technology/human relations by shedding light on the significance of autonomy in such relations. Additionally, the article extends these multidisciplinary studies in its unfolding of the modes of existence at play in AI/human relations.

The article demonstrates how the idea of autonomy sometimes enables relations and at other times is an obstacle in relations. The empirical examples offer insights into how humans and AI build, understand, and engage in their relations. AI/human relations emerge as complex assemblages of nonhuman/human collaboration, negotiation, coordination, and tension—suggesting important distinctions from current AI/human dynamics. For example, AI/human collaboration has proven to be more socially complex than a mere delegation of tasks. I argue that AI/human dynamics are not so much determined by official roles on paper as by the practical relations they engage each other in. Following such results, the article suggests a theory of relationality that redirects the focus from questions of agency to questions of relations and what it means to make relations.

## Nonhumans, agency, and autonomy

Questions of nonhumans, agency, and autonomy have long been debated in social theory, and there has been strong resistance to attributing any kind of agency to nonhumans. These nonhumans, Latour’s (1992) ‘missing masses’, are therefore commonly excluded from accounts of world-making and from social theory. This is evident in the Cartesian tradition, for example, and is articulated by (a) seeing humans and nonhumans as deeply ontologically separate, (b) assuming that any attributes of an entity (human or not) are fixed and reside within the entity, (c) some attributes belong exclusively to humans, and (d) denying nonhumans agency. Such ideas are grounded in human exceptionalism, where we have trained not to be affected by nonhuman others (van Dooren, 2014). As Despret (2016) observes, suggestions that nonhumans ‘do’ certain things that might normally be connected to human characteristics may therefore be hard to accept.

These traditional notions of nonhuman agency have been challenged by scholars in the field of Science and Technology Studies (STS)—specifically in the wake of actor-network theory (ANT) and more recently within multispecies studies. These, but especially the latter, stress the need for more generous approaches to nonhuman/human relations (Descola, 2013) and argue for a reinvention of relations between humans and nonhumans (de Castro & Skafish, 2014). In multispecies work, scholars have shown how all sorts of nonhumans (living and not), such as animals (Despret, 2016; Fudge, 2019; Porcher & Schmitt, 2012), mushrooms (Tsing, 2015), microbes (Schrader, 2012), and other various messmates (Haraway, 2016), mediate action, intervene, object, interrupt, and act in unexpected ways (Despret, 2020). Such analyses challenge prevailing ideas of humans as the sole active subject, exploring how all entities become what they are through entanglements with other beings (Oyama et al., 2001). No entity is autonomous, or self-making. Entangled life depends on connections. Exploring such connections, how AI and humans ‘make kin’ (Haraway, 2015) and what the consequences of such relations are, should be high on the agenda if we are serious about finding sustainable ways of living together. However, in multispecies work, abiotic entities largely remain to be explored. With the broad introduction of AI in our society, humans are set to collaborate with technology ascribed with control and decision-making features, but at the same time reproduce reductionist views of autonomy and agency in AI developments (Suchman & Weber, 2016). Therefore, it is important to further empirically explore assumptions about autonomy in practice and how such assumptions are acted upon. Although it might not make sense for Durkheimian social scientists to approach technology as part of world-making practices, STS scholars have explored how technology might change human actions and social relations (Akrich, 1992; Shapin & Shaffer, 1985), showing the interdependence between humans and technology in practice (Latour, 1996; Law, 2002; Strathern, 2006; Suchman, 2019). In such studies, scholars have also observed the blurry boundaries between humans and technology in situations where they are set to interact (Alač, 2009; Prentice, 2013). Nonetheless, in social theory, the actions of the machine are commonly seen as irrelevant (an argument made some 30 years ago by Woolgar (1991) and still surprisingly relevant). Ascribing ‘autonomous features’ to technology further elevates questions of technology/human relations, pushing the importance of asking anew how they ‘make kin’ (Haraway, 2016).

Proposing a theory of relationality widens the possibilities for action and its complexity, turning attention to relations, rather than agency. The problem with ‘agency’ is that it reinforces the assumption of a single actor behind an action, which risks overlooking the many ways in which action is made possible. If we become what we are together with others (Oyama et al., 2001), then we need to keep in mind the important argument made by Porcher (2014) about the need to always think about nonhumans and humans together. Doing so in the case of AI and humans makes it possible to imagine their relations as being something else than a competitive one.

To approach relationality, Derrida (2008) suggests that we refrain from dividing ‘human *response*’ from ‘nonhuman *reaction*’. If technology is approached as *reacting*, then its interactions with others are easily anticipated. Approaching AI as reacting would thus deny its possibilities to act in unexpected ways and, more significantly, its possibility to affect the relations in which they are part. As we shall see, that is not the case in the explored AI/human relations. The article instead proposes the risky hypothesis that AI *responds* to proposals and changes in its environment, arguing that the idea of AI as a ‘mechanical agent’ does not cover the many ways in which AI makes its relations. By approaching AI as *responding*, we can maintain a focus on what the AI is actually doing and not just what it is supposed to be doing. In practice, exploring AI/human relations, we need to stay curious about what our machines are up to. What is at stake here—politically and ethically—is an expansion of our existing relational imaginary trying to ‘think differently’ (Dahlin, 2023) about AI/human relations.

## AI/human encounters

Every part of the system is as complicated as the system as a whole. Every plate we unfold is itself made up of plates to be unfolded!

(Latour, 1996)

My case is an example of an AI system and humans working in collaboration to carry out a medical procedure. The materials for this article concerning the AI and robotic CyberKnife system were generated through ethnographic fieldwork (Hammersley & Atkinson, 1983/2007). The ethnographic work includes observations of medical staff’s work with the different parts of the CyberKnife system; informal conversations with medical staff and engineers; personal communication via email with the observed medical staff and engineers; technology demonstrations; and information brochures, websites, and International Organization for Standardization (ISO) and the International Electrotechnical Commission (IEC) joint standardization document on medical electrical equipment (IEC/TR 60601-4-1).

Analytically, I practised what Despret (2016) would refer to as ‘thinking with’ other beings, studying how the involved entities (humans and AI) rendered each other capable in actual encounters. Haraway (2008) refers to such actual encounters as ‘contact zones’ where we can learn in what ways AI and humans shape, and change with, each other together. These observations were carried out as a walk-through in the hospital, led by individual medical staff members. During the observations, I made ‘thick descriptions’ (Geertz, 1973) of *how* the interactions between the system and the medical staff played out. The empirical explorations were part of a broader study that included investigations of different AI and robotic systems in medical practice, in an attempt to challenge problems in social theory about nonhumans and agency. The broader study includes fieldwork carried out at teaching hospitals in the UK and the US. Complementing interviews were conducted via email with the observed medical staff (to confirm observations made), and with engineers familiar with the AI and robotic system (to confirm technical functions). Additionally, I did qualitative textual analysis (Silverman, 2015) of the inscriptions surrounding the CyberKnife system (such as protocols, legal documents, academic articles, ethical guidelines, standardizations, and information material). Much of such material was suggested by the engineers involved in the study. The work done by such inscriptions, in relation to the observations and conversations with both medical staff and engineers, supported the analysis.

Now, let’s meet our friends for this expedition. First, the CyberKnife system is a robotic radiosurgery device that plans and carries out radiation treatment to kill cancer cells and shrink tumours, and for treating other conditions where radiation therapy is indicated. What differentiates the CyberKnife system from conventional radiation therapy is that it delivers radiation to the target in small doses from a multitude of different positions. This way, the ‘beams of radiation can be nonisocentric to achieve high conformity and allow for a steep dose gradient around the target’ (Gerlach et al., 2020, p. 3806). This complex system of both hardware and software includes different devices such as a ‘stereoscopic X-ray system with in-floor detectors’, a ‘respiratory camera’, a ‘robotic arm’, a ‘treatment couch’, and a ‘robotic collimator changer’ (CyberKnife, 2023). It is an example of rapidly evolving applications of deep learning models and robotics in medical practice.

Second, alongside the CyberKnife, we will follow medical staff (radiologists, a medical physicist and an oncologist) working with different parts of the system. A couple of engineers will also show up as the story unfolds. They arrive in the company of inscriptions such as ISO standards, protocols, and other information material about the CyberKnife system. To ensure anonymity, all characters except the CyberKnife system have been given fictive names. While the scope of this text will not allow us to fully explore the complex system that is the CyberKnife, a few things need to be said about it to familiarize ourselves with one of this story’s main characters.

Even though its name might indicate that there is cutting involved, the CyberKnife system does not involve any knives. The treatment that the system carries out is often described as radiosurgery. The term ‘surgery’ here refers to the precision of the delivery of radiation beams rather than excision with a knife. Since the CyberKnife’s first treatment of a patient in 1994, the system has continuously been developed (Pollom et al., 2019). The manufacturer, Accuray, describes this system’s principles as:

The only robotic radiosurgery system that can deliver non-surgical stereotactic treatments with sub-millimetres accuracy, anywhere in the body—including the prostate, lung, brain, spine, liver, pancreas and kidney. The world’s only motion-synchronized, AI-driven, real-time treatment delivery adaptation for all indications and treatments.

The medical staff’s work with the CyberKnife can be divided into two main parts: first the *treatment planning* (which involves a software programme), and then the *treatment delivery*, which involves both software and hardware. The different parts of the CyberKnife system are physically dispersed, spread out across different rooms and different floors within the hospital. It is now high time for us to delve into our empirical stories. In what follows, I focus on interactions between the CyberKnife and its collaborating humans.

## Some notions of autonomy

Reading up on the CyberKnife before I started fieldwork, I found narratives where the term *autonomous* was frequently used when referring to the CyberKnife system (Eldin Abdelaal et al., 2020; Ficuciello et al., 2019; Haidegger, 2019a, 2019b; Oh et al., 2016; Troccaz et al., 2019; Yip & Das, 2017). As described by Accuray, the manufacturer of the CyberKnife system, the ‘radiosurgery delivered by CyberKnife is autonomous but delivered under human supervision’ (Kilby et al., 2020, p. 16). I also learnt that classifications for evaluating the actions of AI systems in medical practice divide different systems into different *degrees of autonomy* (DoA) and different *levels of autonomy* (LoA) (Haidegger, 2019a). In such classifications, the CyberKnife system is, as the only system, placed just below the highest *level of autonomy*, where systems are described as having the ability to:

Execute complete procedures based on human-approved surgical plans, while the human only has the capability to emergency stop the procedure. The robot shall be able to complete the task even if the human fails to respond appropriately to a request to intervene. (Haidegger, 2019a, p. 71)

No surgical system has yet qualified for full autonomy, according to Haidegger (2019a). Systems that would qualify for the highest level of autonomy that the engineers have set up are described in the following way: ‘The system succeeds in scenarios where even the best human operator would fail, therefore there will be no need for a human fallback option’ (Haidegger, 2019a, p. 71).

As we enter the hospital, we find ourselves at the Cancer Centre of our teaching hospital. On the second floor, in a room full of computer screens, we find John, a medical physicist. The robotic hardware device that will later deliver the treatment to a patient is on a different floor. One of the computers in John’s room has a Cyberknife software programme installed on it. At the end of the work carried out in this room there will be a treatment plan for the robotic hardware device to carry out. But before John describes and shows me his work with the software, he has something he wants to clear up. John and I had been in contact via email, and now he wants to set the record straight:

In your email, you referred to the CyberKnife as autonomous, and I thought that was a bit strange. You see, we tell the machine what to do. It does not do anything on its own. We are the ones doing everything. It just follows the plan that we programme it to carry out.

Before I had time to beat myself up any further over my choice of words in that email (which by the way, since we are delving into terminology, was ‘how such tasks—that the CyberKnife carries out—could be described as autonomous to some extent’), John continued: ‘I do not think that the CyberKnife is autonomous. It is just a machine over which we people are in total control.’

This event drew attention to the complexity that the term *autonomy* brings. John continued to describe the CyberKnife as *not autonomous* since, as he said, ‘it does not make any decisions about how the treatment is delivered’. That I had referred to the system in this way in my contact email kept bugging John, as I could tell because he kept bringing the matter up.

John is not alone in his objections. During fieldwork, I came across different situations with various medical staff, engineers, and AI developers in medical practice where terminology was a source of tension. In fact, discussions over terminology occurred frequently. A significant example concerned a meeting where international guidelines for technological innovations containing AI was to be formulated. I was told that it was difficult for those involved to agree on terms such as *machine learning*, *autonomy*, and *artificial intelligence*, and what each entails. The different experts spent much of the meeting discussing what terminology to use in the guidelines and how to define each concept. The concept of autonomy proved to be far from self-evident. Nor does it become any more clear when we take a closer look at how autonomy is managed in practice. As we soon shall see, nonhumans are more easily suspected of lacking autonomy than humans are (Despret, 2016).

## Ascribing/denying

Let’s return to our hospital where John is working on a treatment plan for a patient. He describes how it can take up to several days to make a treatment plan, depending on the complexity of the treatment. He explains: ‘The system can make use of 120 different positions around the patient from which to deliver beams, and from any position, beams can be delivered in different angles.’

That is what a treatment plan is, then—an arrangement of radiation beams to be delivered through a geometric choreography. That is, as John explains it, the task of the CyberKnife system in the treatment planning is to generate a beam arrangement that delivers the dose of radiation to the targeted tumour. But to get there, more work is required.

First, he says, the CyberKnife system needs information to be able to decide how to deliver the treatment. Before a patient receives treatment, they come in for X-ray scans. John explains to me that these scans are incorporated into the CyberKnife’s software system, which then generates three-dimensional images of the area where the tumour is located. The software system uses the scans to determine the size, shape, and location of the tumour and construct a virtual, three-dimensional model of the tumour. To do this, John explains that the system needs information about the patient in the form of the scans as well as information about the relations of different structures and tissues. Furthermore, John says, what information the system needs depends on where the tumour is located in the patient and the shape of the tumour. Generating these three-dimensional images of the X-ray scans is the first task that needs to be carried out.

On his computer screen, John shows me a treatment plan for a patient who is soon going to have radiosurgery with the CyberKnife system. John explains:

So, what I do is I tell the system what I want [John refers to this as ‘objectives’] and what I don’t want [John refers to this as ‘constraints’]. Sometimes we have to build the plan slowly, find a solution, and then start to tune by adding more and more constraints allowing the system to push the dose somewhere else. Think of the dose lines [the radiation beams and their strength] like a balloon squeezing; it is only possible if you allow the dose to spill out somewhere else. The system has a predefined number of beams available, and it considers all the beams to find the best solution. The system needs to take into consideration different tissues [like organs and bone] when finding a solution.

I sit next to John as he shows me images of a brain on his computer screen. Holding a pen to the screen, John points out where the tumour is. As he follows the edge of the tumour, he explains: ‘This is where we want it to work.’ While John is describing the details of creating a treatment plan, the oncologist, Mary, comes in. She and John start to discuss the scans on the screen and talk technical details, pointing to the screen with their pencils and the mouse pointer. When Mary leaves the room, John continues: ‘The system calculates and determines every radiation beam—their angles, shapes, and the dose of radiation that will then be distributed to the treatment area.’

Look at that—the CyberKnife got its autonomous features back! Describing the back-and-forth collaboration between himself and the system when making a treatment plan, John describes the system as considering things, finding a solution, working, and calculating. Moreover, John is not alone in describing the system’s actions this way.

Leaving John, I walk with one of the radiotherapists, Anna, down to the room where the patient soon will get radiation therapy delivered by the CyberKnife. As we make our way through the hospital we are accompanied by a sharp smell of disinfectant and cleaning agents, a lingering stubborn antiseptic breeze. The fluorescent lamps in the ceiling don’t leave much to the imagination. We pass numerous booths with curtains in a washed-out blue fabric that does its best to offer a sense of privacy to the patients placed between them. The control room is the place from which Anna and George, another radiotherapist, monitor the treatment. It is placed right next to the area we have just passed, where patients were waiting to get their treatments. The control room is a small, dark room with two desks, a couple of chairs, and several computer screens. It is placed right next to the treatment room where the CyberKnife robotic device carries out the radiosurgery. George explains: ‘The CyberKnife will carry out the treatment plan when we run it. It would not even know if we had an actual patient in there or not.’ And just like that, the CyberKnife has, once again, lost its autonomous features.

Through a large window in the control room, we can see the treatment room where the CyberKnife robotic device is placed. There is a heavy door dividing the control room from the treatment room. Both the CyberKnife and its humans have their own workspace during a treatment. All the CyberKnife’s human co-workers are required to leave the treatment room before the treatment can start, to limit human exposure to radiation. To deliver radiation treatment, the CyberKnife needs to be alone with its patient. Anna describes the radiation therapists’ work when the CyberKnife robotic device is delivering radiation therapy to a patient. She explains that she follows the treatment on computer screens here in the control room:

We make sure that the CyberKnife is treating the exact right area where the tumour is located. We follow where in the treatment plan the CyberKnife is. It’s like when you play a song and you can follow where in the song you are, at any given moment, by following the timeline. Other things can also impact the tumour’s location, for example if the patient coughs, or fluids and gasses, and the radiation beam would be delivered to another area than planned had the CyberKnife not been able to follow the tumour’s movement in real-time. However, sometimes the CyberKnife wanders off and acts on its own.

Once more (!) the CyberKnife is back on the social scene. What the CyberKnife is, however, changes as George and Anna go on. Let’s move into the treatment room.

The CyberKnife begins moves into a specific position, stops, delivers a radiation beam, and then moves into another position to deliver the next beam. Next to the robot is a table holding a range of collimators, devices for shaping the radiation beam to deliver the correct dose of radiation. The robot picks up a collimator, uses it, and then exchanges it for another one. The CyberKnife moves around the patient and, from different angles, delivers radiation beams at the same time as it continuously calculates to verify the location of the tumour. This continues until all beams from the treatment plan are delivered. George explains that the radiologists can pause the programme if they need to. Anna again:

The CyberKnife can mistake a scar for a lesion and then wants to treat the scar instead. If this happens, I can take the live images and use the computer mouse to drag the live images over the images from the treatment plan. By dragging the image that the CyberKnife is taking during the treatment back over the images in the treatment plan, we tell the CyberKnife that this is the area you should concentrate on. The CyberKnife understands this.

What autonomy is and how it is understood differs from situation to situation. That is, autonomy was not self-evident, but unstable and situated (Haraway, 1988). Looking more closely at the details in the practices where our humans and the CyberKnife system worked closely together, they ascribed different attributes to one another at different times (Haraway, 2018; Latour, 2014). Our humans were willing to sometimes ascribe to the CyberKnife autonomous features commonly attributed exclusively to human actors (Despret, 2016), by describing it, for example, as something that could *learn* things, *consider* things, *find solutions*, *calculate*, *determine*, *understand*, *work*, and *act on its own*. However, cut to a different situation and our humans are equally eager to deny the CyberKnife any autonomous features. Remember an earlier statement from John, that it is the humans involved who are ‘doing everything’, denying it any kind of autonomous features. While this echoes Woolgar’s (1991) reasoning that the actions of machines are commonly seen as irrelevant, it also exposes the medical staff’s ambivalent views about the actions of the machine.

One of the questions I asked John was whether he had ever experienced the CyberKnife system making a mistake, or if he himself had ever experienced something going wrong. John’s quick response surprised me. Almost before I had even finished the question, John replied without any hesitation.

No, never heard of it. I never even heard of the CyberKnife making any mistakes or errors of any significance. There was an event in France a couple of years ago when something went wrong, but that was connected to the programming. It was a human error. The robot never makes mistakes.

This resonates with common narratives of AI and other technologies, which is to blame the operator, the human actor, rather than the technology (Koopman, 2018; Stilgoe, 2017; Tennant & Stilgoe, 2021). However, we could also see that medical staff often demonstrated a reluctance and uneasiness toward machine agency in line with human exceptionalism (van Dooren, 2014) and reductionist logics (Haraway, 1991; Suchman & Weber, 2016), which affected how they thought about and perceived autonomy and nonhuman agency.

As we could see, the humans in our story continuously switched between registers, sometimes ascribing autonomous features to the system, and sometimes denying it such features. Whether or not the system was rendered autonomous depended on the situation. In specific situations and regarding specific tasks, it was sometimes important for medical staff to relate to the AI as if it had autonomous features. In other situations, ascribing such features to the AI raised concerns and became problematic. In these situated notions of how to understand the machine’s actions, the AI system acted as a forceful model for resisting reductionist logics.

## Enabler/obstacle

We have now seen how autonomy can be a sensitive issue. In some situations, humans were more willing to attribute autonomous features to the system than in others. It proves to be even more complicated during some events where tasks were delegated and coordinated between AI and humans in practice. In these situations, it seems unclear who is responsible for which action, exposing the blurry lines between humans and machine (Alač, 2009; Prentice, 2013). Let’s return to our friends at the hospital and see what happens in practice when the system is sometimes ascribed autonomous features and made into an enabler of work, and sometimes denied such features and turned into an obstacle in work.

Anna and John have different training and do different work with our CyberKnife. Each does their part in accomplishing radiation therapy, but neither has a complete overview of the work with the CyberKnife system. John works with the system in creating a treatment plan, and Anna in treatment delivery. When Anna is handed a finished treatment plan by John, she takes it to another room, one floor down. In Anna’s words: ‘When there is a finished plan, I run and supervise the treatment. I don’t check or control the plan. I just run it.’

In the treatment room, a large area on the floor is painted in a contrasting colour, purple instead of the grey of the rest of the floor. Anna explains that the coloured area is the space in which the CyberKnife can move. She continues:

From these screens, we can monitor the treatment plan and follow where in the treatment plan the CyberKnife is. Before the treatment starts, we prepare by placing the patient on the treatment couch. We then leave the room and run the treatment plan. And these four screens are connected to the CCTV in the treatment room. From these screens, we can monitor the patient being treated. There is also an intercom so that we can communicate with the patient during their treatment. We can decide how often we want the CyberKnife to take live images during the treatment. How often we want it to take images depends on the patient’s individual treatment plan. It depends on the complexity of the treatment plan.

The CyberKnife system also monitors itself during the treatment through real-time image guidance. Moving into the treatment room, there are two cameras that are part of the CyberKnife system that repeatedly take images during the treatment. Pointing to the ceiling, Anna explains, the CyberKnife continuously compares those images to the images from the treatment plan. The CT scans from the treatment plan create a three-dimensional map over the tumour and the area to which the CyberKnife system is going to deliver its radiation beams. To exemplify, George, the other radiotherapist in our story, presents an event where the patient getting treatment has a lung tumour:

Since the patient is breathing, the location of the tumour keeps changing. The tumour is constantly moving. The CyberKnife needs to know, at any given moment, exactly where the tumour is when delivering its beams. To do so, the system tracks and follows the patient’s breathing patterns following the tumour’s motions. This way, the CyberKnife can treat the tumour while it’s moving.

The scans that are taken before the treatment are used to create a series of images that capture the individual patient’s complete respiratory cycle (Urschel, 2007). This model of the patient’s breathing pattern is then continuously compared and updated with the images taken during the treatment by the optical cameras in the treatment room, which correspond to X-ray imaging detectors on the floor. Markers (in the form of LED) are placed on the patient’s body to compare the breathing pattern from the scans to real-time patterns.

The radiation therapists oversee the treatment to make sure nothing goes wrong. But how can they be sure that everything is going according to the plan and that the CyberKnife is doing its job correctly? They cannot see for themselves *how* the CyberKnife treats the tumour by observing the treatment room. No, they must rely on their computer screens in the control room, where they can follow the CyberKnife’s work by looking at scans and following where in the treatment plan the CyberKnife is currently operating. Listen to what John has to say about the work of monitoring the CyberKnife:

I would not feel safe if the CyberKnife were not just running a programme and if it was not for all the people involved in all the steps of the treatment with the CyberKnife. We are constantly overviewing everything and we tell it what to do. It would not feel safe if we did not control the CyberKnife as we do.

Did you hear that, dear CyberKnife? You merely run a programme; the humans tell you what to do. I guess you are off the social scene again.

To our medical staff, it matters if it is a human or a machine who is in control or making certain decisions in specific situations. Ascribing autonomous features to the system raises concerns and is described as an obstacle in some situations. Here, we could see how imaginaries about humans and machines (grounded in traditional dualistic thinking) play a role in how humans behave around AI systems, and how they understand autonomy in certain situations (Suchman & Weber, 2016). The stories bear witness to how humans assume that they make ‘better’ or ‘safer’ decisions and are more trustworthy when it comes to being in control. This also manifests a resistance to acknowledging machine decision-making. At the intersection of these various AI/human encounters we could see how autonomy was challenged, negotiated, and reproduced in different ways in various situations.

Different aspects of monitoring seem to be involved in the treatment delivery. Let’s sum up the ways in which radiotherapists Anna and George monitor the treatment delivery, and perhaps we can clear up a few things in terms of their relations with the CyberKnife. As radiologists, they (a) monitor where in the treatment plan the CyberKnife is at any given moment, (b) monitor how many live images the system takes, (c) monitor the treatment delivery via the CCTVs, and (d) monitor that the CyberKnife is treating the right area (if the CyberKnife is not treating the right area, they tell the system where it is supposed to treat by using the computer mouse to draw the live X-ray images over the X-ray images in the treatment plan).

But wait a second—the medical staff were not the only ones monitoring. Just a moment ago, it was the CyberKnife system that continuously monitored itself through live X-ray images. The medical staff explained in detail how you, dear CyberKnife, keep track of the precise (and possibly moving) location of the planned treatment area that is the tumour. It looks to me as you, dear the CyberKnife, and your collaborating medical staff share the responsibility of monitoring, making monitoring a collaborative effort in which they depend upon, and are guided by, each other. Such interdependent monitoring provokes the question, in practice, of who is working for whom and who is in control of which tasks. The emphasis on monitoring, carried out by medical staff as well as by the CyberKnife system, draws attention to how advanced technology systems challenge established views of the role of technology—the classic old settlement of ‘humans as active subjects’ and ‘technology as passive objects’ (Latour, 2005). Back on the social scene once again, my friend!

We have seen how ascribing autonomous features to the system could be an obstacle when it comes to being responsible for monitoring the treatment delivery. However, sometimes ascribing autonomous features to the system can enable work. Let’s go back to John and the production of a treatment plan. The images on John’s computer screen are in black and white. When the software has made its calculations and suggests a treatment plan, one can see all the beams in the images in different radiant colours, cutting through the treatment area from different angles. The different colours of the radiation beams signal how much radiation each beam contains, John explains.

A treatment plan has now been generated. However, sometimes more work is required. For example, the system can suggest that radiation should be delivered through sensitive tissues or organs that the medical staff do not want to treat with radiation. John describes what happens:

The planner [this is what John calls himself when working with the software system] needs to tell the system what he wants. This can be done by creating a ‘copy’ of the tumour—a planning tumour. The system is then presented with a new image where the difficult area that we do not want to treat is cut off. This is a way to tell the system that this is the tumour we want to treat. The system then generates a new plan, based on the planning tumour instead, now without damaging sensitive tissue. This way, I can teach the system how to work around the problem. By offering the system a planning tumour, I can manipulate the strength of my demands.

Oh, I can hear you from here, dear CyberKnife: ‘Poor humans, cannot even do their own calculations.’ But John has some tricks up his sleeve. According to John, it is up to the system to find a solution that meets the requirements that the planner sets up. John cannot find a solution himself. He cannot just push a few buttons and correct mistakes or generate a treatment plan all by himself. The CyberKnife does not allow for that kind of action to take place. Instead, he depends on the CyberKnife’s software system to do this. John’s detailed description articulates the chronological work of a careful choreography (Cussins, 1996) that he and the system accomplish together. What is expected of John and the CyberKnife is that their cooperation will result in a treatment plan. This is necessary for radiosurgery to take place. So how does John move forward?

John tries to find a way to communicate with the system so that the system can then generate a plan closer to what John has in mind. That is, the planner needs to find a way to tell the system what he wants. He engages in what Haraway (2008) would describe as ‘practices of communication’, ascribing to the CyberKnife an ability to understand. Moreover, John not only communicates with the system, but also negotiates with it. John imagines how the system works and how it will respond to his propositions. That is, to understand the CyberKnife, he imagines how the CyberKnife would respond to different kinds of actions (Hutchins, 1995). He imagines what the system wants. This way, the system makes the job of the planner possible. John not only makes himself available to the system and shows an interest in understanding it, in the process of doing so, he changes.

In specific situations and regarding specific tasks, it is sometimes important for medical staff to relate to the AI as if it has autonomous features. That is, for some tasks they rely on the CyberKnife to be able to do things on its own. This episode demonstrates that both John and the CyberKnife system are engaging each other in communication of some sort (Despret, 2005; Porcher & Schmitt, 2012). They both improvise the work depending on the other’s response. John portrayed the work of producing a treatment plan as a sort of negotiation between himself and the system, in which he handed over certain tasks, control, and decisions to the system. John also described how he attuned himself to the system to make himself understood. In such situations, the CyberKnife was an actor involved in the work process. On the other hand, when John is asked who made the treatment plan, there is no hesitation at all—he made the plan.

Autonomy may sometimes be what is seen as enabling collaboration, and at other times it is an obstacle in the collaboration. The scenes explored above reveal that humans’ understanding and perception of who was in control or responsible for decision-making in a specific situation were based on their experience of the situation at hand rather than on their official roles. That is, the AI system and the humans did not follow a specific delegation of tasks determined beforehand. The question of who the humans deemed responsible for what actions was much more complicated than that, and was shown to be attached to complex understandings of what autonomy is. If only the CyberKnife could tell their version of the story!

## Conclusion: Dancing around autonomy

In this article, I have continued debates in STS on analytical symmetry toward technology/human relations, turning to the significance of autonomy in such relations. By connecting such debates with insights from multispecies studies on how nonhumans and humans make their relations, I turned attention to situations in which AI and humans connect, exchange their properties, transform and affect each other in unexpected ways. These are situations in which technology and humans ‘make kin’ (Haraway, 2018), and what Latour (2017) would refer to as ‘metamorphic zones’. The explorations of the case of the CyberKnife offer empirical and theoretical insights into AI/human dynamics. The article posed the question of the significance of autonomy in AI/human relations and how autonomy might change or challenge technology/human relations.

The empirical analysis shows that the very idea of autonomy is a double-edged sword. We can see how the humans sometimes ascribed certain autonomous features to the AI system, whereas in other situations it was denied such features. For example, the humans sometimes described the system as something that could learn things, understand things, find solutions, calculate, determine, work, act on its own, or even wander off. At other times, however, it was the humans who were responsible for all such actions. Moreover, it was *sometimes* important for medical staff to relate to the AI as autonomous, describing its autonomous abilities as enablers of the work carried out. In other situations, such abilities were considered a risk, and the AI system then became an obstacle in the collaboration. That is, for the humans in our story, it mattered whether a specific task was carried out by a machine or a human.

The accomplishment of radiosurgery was not the sum of only medical staff’s actions. For example, we saw how medical staff steered the actions of the CyberKnife, but also how medical staff’s actions were steered by the CyberKnife system. Although the CyberKnife system and its collaborating humans from time to time handed over work to each other (a task, control, decision-making, or problem-solving), it was not necessarily clear in specific situations who was responsible for what. At least not to our humans. While there was uncertainty over responsibility in the AI/human relation, our friends at the hospital did not express concerns or complaints about this. Both AI and humans (together with others) shared work, coordinated different tasks with each other, handed tasks back and forth to each other, negotiated, and collaborated, and in the process of doing so they conducted radiosurgery. However, in such work, the humans in our story also coordinated their perceptions of autonomy. This sheds light on the shifting significance of autonomy that humans establish as they negotiate roles in practice. Furthermore, it reveals how autonomy came to matter less as a fixed category than as a situated one. This points to the importance of learning how to, in Haraway’s (2016) terms, ‘stay with the trouble’ regarding how humans think about, articulate, and act around autonomy in practice.

Thinking of AI as *responding* opens up possibilities for a more nuanced analysis of technology/human relations. Despret (2016) recommends that this more generous approach to nonhuman can work to ‘provoke a hesitation’ about conventional thinking regarding the roles played by specific nonhumans. This, in turn, can help us rethink our relations with AI and reshape our ‘sociotechnical imaginaries’ (Jasanoff & Kim, 2009). Debaise and Stengers (2017) would call this engaging in ‘speculative thinking’ about the capabilities of technology. Such an approach to the study of AI systems’ parts in AI/human collaborations opens up new possible ways of interpreting the AI’s actions and thereby opens up for new ways (and more ways) of knowing our machines—learning new modes of relating. Such thinking offers a wider conceptual apparatus that increases rather than reduces the possibilities for action. Such approaches seem necessary if we want to learn how to live better with AI in the future.

In this study, radiosurgery-with-AI was made sustainable through a mess of handover moments, with actors moving between different versions of themselves and others, and distributing different attributes to each other at different times. This movement generated the relationship constellation and in turn shaped and produced the practice in which both humans and the AI system were engaged. Such ongoing relating is always temporary and never conclusively established (Stengers, 2011). This, in turn, draws attention to the kinds of interspecies (organic and otherwise) dynamics that shape AI/human relations. The empirics thereby defy simplistic explanations and highlight the complexity of making relations with AI machines. Rather, the empirics showed how AI systems act as figures of technology, neither in control of nor being controlled by humans, but rather challenging and resisting such reductionist logics. AI/human relations are thus not so much determined by any ostensive delegation of tasks as by how humans and technology make their relations in practice.

Taking into consideration that humans switch between registers and coordinate work around their shifting orchestration of autonomy, the very notion of autonomy can be seen to be multifaceted. As the development of AI technology problematizes terms such as *autonomy* in human/machine constellations, this article emphasizes the need to move from the idea that ‘agency’ is attributed to a specific subject or object in a specific situation. The interactions between our humans and our machines proved to be much more complicated than that. This illuminates the need for a theory of relationality that redirects focus from questions of agency to questions of relations and what it means to make them.

## And as for you, dear CyberKnife

In Latour’s (1996) story of the transportation system Aramis, the technology was not in use nor even yet fully realized. And here you are, dear CyberKnife system, in use, working, collaborating with others, but still there are as many interpretations of you as of our old friend Aramis! I am sorry that I put you in this awkward position by starting to talk about you as having *autonomous* features. If it is of any comfort to you, I put myself in this horrible situation too. The idea of autonomy is indeed poorly thought through. I really should have known better. No being is self-made. You are of course *symbionomous*—like the rest of us!
